# The Effect of Mindfulness Intervention on the Psychological Skills and Shooting Performances in Male Collegiate Basketball Athletes in Macau: A Quasi-Experimental Study

**DOI:** 10.3390/ijerph20032339

**Published:** 2023-01-28

**Authors:** Yan Wang, Si-Man Lei, Chi-Chong Wu

**Affiliations:** Faculty of Education, University of Macau, Taipa, Macau SAR 999078, China

**Keywords:** mindfulness, acceptance, anxiety, basketball shooting performance, intervention

## Abstract

Background: This study adopted a quasi-experimental design to examine the effect of a 7-week mindfulness intervention on the psychological coping ability and shooting performance of college-level male basketball athletes in Macau. Methods: A total of 43 male college basketball athletes in Macau were selected as the participants. Besides the regular basketball training, the intervention group (*n* = 23) received a 7-week mindfulness training; the weekly mindfulness intervention session lasted around one hour according to the mindfulness training manual for athletes, while the control group (*n* = 20) did not receive any mindfulness training. Before and immediately after the 7-week intervention, all players performed the following tests: the “Five-Facet Mindfulness Questionnaire”, the “Acceptance and Action Questionnaire”, the “Sport Competition Anxiety Test”, the “Mindfulness Attention Awareness Scale”, and three shooting tests. An independent-sample t-test and a paired-sample t-test were used to analyze the between- and within-group differences. Moreover, a repeated measures ANOVA was used to assess the group, time, and group-by-time effects on psychological skills and shooting performances. Results: The intervention resulted in both significant between-group and within-group differences in mindfulness level, acceptance level, attention level, three-point, and free-throw shooting performances (all *p* < 0.05, Cohen’s d ranging from 0.565 to 1.117). Conclusion: While further study is necessary, the present study suggests that the 7-week mindfulness training program can significantly improve psychological outcomes and shooting performance in Macau college basketball athletes. Future studies involving competition settings and objective metrics will aid in verifying mindfulness as the prevalent practice among basketball practitioners and athletes.

## 1. Introduction

Basketball has a large fan base and high popularity all across the world. As the level of competition in basketball has risen over the years, there is a greater need not only for the athletes’ physical ability and technical skill, but also for their superior and consistent psychological skills [1]. With the rise of performance psychology (a subdivision of psychology that examines psychological factors influencing optimal human performance) [2], the application of psychological training approaches to increase sport performance under pressure conditions is a major focus for sport psychologists. Over the last 40 years, traditional psychological skills training has been widely used in sport and performance psychology to assist athletes in improving their performance. The psychological skills trainings are a group of mental adjustment techniques (e.g., imagery, positive self-talk, goal setting) emphasizing self-control of internal processes, such as thoughts, feelings, and bodily sensations [3,4]. Numerous research findings showed that psychological skills training may be successful in improving those psychological characteristics theorized to be associated with athletic performance; nevertheless, no substantial changes in athletic performance were discovered [5,6,7].

Given the limitations of traditional psychological skills training, additional ways to enhance performance are required. Mindfulness is drawn from the Eastern Buddhist concept. In psychological applications, it was generally characterized as purposefully paying attention to the present in a nonjudgmental manner [8]. The psychological mechanisms of the benefits of mindfulness training can be mainly summarized as improvements of (1) attention, (2) experiential acceptance, (3) negative emotional regulation (e.g., anxiety and fear), (4) psychological flexibility, and (5) non-attachment and less rumination [9]. In recent decades, the use of mindfulness-based intervention has gradually gained attention throughout the world and has played an important role in boosting nonjudgmental awareness and acceptance of in-the-moment cognitive, affective, and sensory experiences as well as sports-performance-related outcomes [10,11,12].

Recently, Mindfulness–Acceptance–Insight–Commitment (MAIC) training has gained popularity in interventions among people from Eastern cultural backgrounds [13]. MAIC is based on Eastern traditional cultural ideas, combined with the concept of adversity coping and Zen enlightenment [14]. Moreover, MAIC innovatively involved two new elements, “value” (broad preferences concerning appropriate courses of actions or outcomes) and “insight” (new awareness or discovery of life), into the training to make it more adaptive and acceptable for Chinese athletes [15]. A series of follow-up MAIC intervention studies targeting athletes of dart throwing, shooting sports, and swimming showed that MAIC training could significantly improve their psychological outcomes (mindfulness, acceptance, flow state, anxiety reduction, training, competition satisfaction, etc.) as well as their athletic performance [16,17,18,19].

However, existing research on mindfulness in promoting athletic performance also has certain problems. Despite the growing amount of mindfulness intervention in the field of sport, only a few examined the effect of mindfulness training in basketball. The only limited previous evidence suggested that practicing mindfulness can improve basketball athletes’ free-throw performance [20,21]. In the present era of small-ball, where most offensive options are on the perimeter and quick transition, basketball has evolved into a sport that requires a high percentage of the mid-range shot and the three-point shot [22].

According to various sports reports, some notable basketball players and coaches, including Michael Jordan, Kobe Bryant, LeBron James, Phil Jackson, and Steve Kerr, have publicly discussed their mindfulness meditation practice and its positive impact on mood regulation and shooting performance, and practicing mindfulness can effectively improve concentration, reduce competition anxiety, and make players’ free throws more accurate [23,24,25]. However, research regarding the effects of mindfulness practice on basketball-related psychological and shooting abilities is limited and such effects require more empirical evidence to be validated. To close the aforementioned research gap, the present intervention study adopted a quasi-experimental design to explore the effects of mindfulness intervention on college-level basketball athletes’ psychological coping ability and shooting performance.

## 2. Materials and Methods

### 2.1. Participants and Procedures

In this study, participants were recruited by contacting their coaches. Prior to the study, informed consent was obtained from relevant coaches and players. 50 basketball athletes from two universities in Macau were recruited, including 25 from University A and 25 from University B. The inclusion criteria of the subjects in the study include: (1) college basketball player, (2) male, (3) no previous experience with mindfulness, and (4) no injury problems. No particular criteria regarding training years and athletic level were set. Excluding 7 athletes who could not participate in training due to personal reasons, a total of 43 participants were finally recruited and completed all intervention sessions and tests. All athletes voluntarily participated in this study, and they were fully informed that they had the right to withdraw from the study at any time, and their confidentiality and anonymity of their data would be protected and respected.

The study protocol has been prospectively registered on Chinese Clinical Trial Registry (ChiCTR2200056640). A quasi-experimental single-case design was employed in the study including three phases: (1) a 2-week baseline phase in which self-reported measures regarding mindfulness, acceptance, and anxiety, along with shooting tests including free throw, mid-range shot, and 3-point shot were implemented; (2) the 7-week intervention phase: a 7-session one-hour Mindfulness–Acceptance–Insight–Commitment training program was provided each Friday night between 4 March 2002 and 15 April 2022; (3) the post-intervention phase, which was conducted after intervention phase. Self-reported questionnaires and shooting tests that are same as the baseline phase were provided again for athletes. All questionnaire measurements and shooting tests were performed by the researchers and a trained research assistant. In addition, all questionnaire assessments were conducted in the classroom before the day’s training, while the shooting tests were conducted in the court after the day’s training.

**Figure 1:**
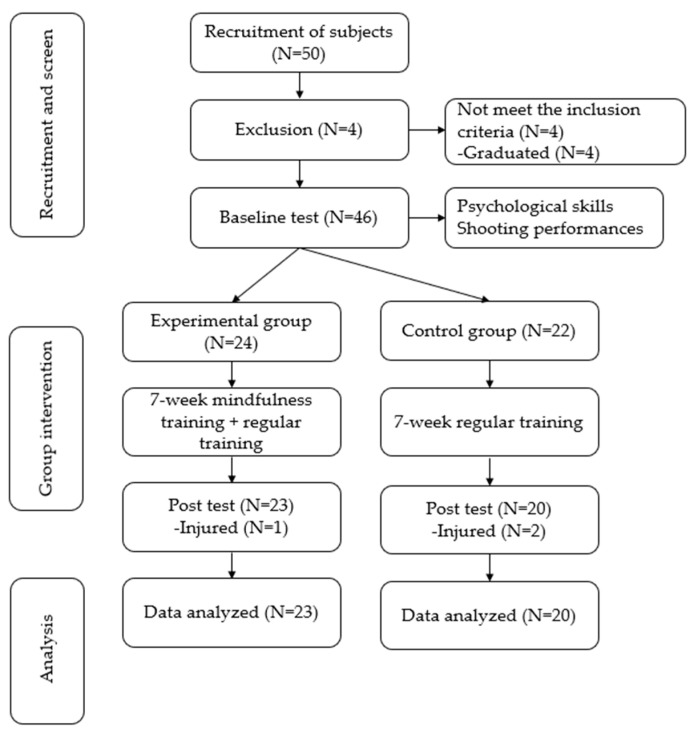
Study flow chart.

Since the intervention was group-based, 23 athletes from the same university basketball team were assigned to the experimental group and received the mindfulness intervention in addition to one of the regular daily basketball practices, while 20 athletes from another university basketball team were assigned to the control group and only engaged in regular daily basketball training without mindfulness or psychological practice. For the convenience of intervention implementation, the mindfulness training intervention program was primarily administered in the university basketball training court and classroom by the researchers who have been trained as instructors of the mindfulness program. The details of the research flow chart are presented in Figure 1.

The intervention material and procedures were mainly adapted from the content of the “Mindfulness Training Manual for Athletes” developed by Si et al. (2014) [26]. The 7-week intervention courses included: (1) preparation of mindfulness training; (2) basic concepts in mindfulness training; (3) self-decentralization training; (4) acceptance training; (5) value and insight training; (6) commitment training; and (7) comprehensive practice. The detailed content of each intervention session is presented in Table 1.

### 2.2. Outcomes and Measures

#### 2.2.1. Demographics and Basic Athletic Background

Basic information regarding age and training experiences (e.g., years of training and training days per week) were collected by questionnaire.

#### 2.2.2. Level of Mindfulness

The Five-Facet Mindfulness Questionnaire (FFMQ) was used to measure the mindfulness level of athletes [27]; it consists of five dimensions (observing, describing, acting with awareness, nonjudging of inner experience, and nonreactivity to inner experience), totalling 39 items (sample item: “While walking, I am aware of the sensations in my body.”), using the Likert 5-point scoring method, from 1 point (“never or very rarely true“) to 5 points (“very often or always true”). FFMQ includes 19 reverse scoring items; the higher the total score of the scale, the higher the level of individual mindfulness, and vice versa. The revised and translated Chinese version of FFMQ validated by Deng et al. (2011) was used in this study.

Deng et al. (2011) translated and revised the FFMQ scale into a Chinese version and concluded that the internal consistency reliability of each subscale was 0.746, 0.843, 0.797, 0.659, and 0.448, respectively, and showed acceptable structural validity in college students and athletes [28].

#### 2.2.3. Mindful Attention Awareness Scale, MAAS

The Mindful Attention Awareness Scale (MAAS) is a 15-item self-report survey that measures the tendency to be fully aware of one’s experience in the present moment without distraction or forgetfulness [29]. Participants indicate whether they frequently or infrequently experience each item using a 6-point Likert scale from 1 (“almost always”) to 6 (“almost never”). The scale was developed with the understanding that people likely have better conscious access to information about their tendency to be mindless rather than mindful. As a result, the total score for the MAAS is computed by reverse scoring and then summing all items. Examples of items include the following: “I find it difficult to stay focused on what’s happening in the present,” and “I do jobs or tasks automatically, without being aware of what I’m doing”. Previous research demonstrated that the MAAS is a sound measure of trait mindfulness with acceptable reliability and validity in a sample of Chinese college students [30].

#### 2.2.4. Acceptance and Action Questionnaire—Second Edition, AAQ-II

The experience acceptance and experience avoidance tendencies were measured by AAQ-II, which is a one-factor 7-item 7-point Likert scale from 1 (“never true”) to 7 (“always true”) [31]. Higher total scores indicate greater levels of psychological inflexibility. Zhang et al. (2014) translated the AAQ-II scale into a Chinese version and validated it (sample item: “My painful experiences and memories make it difficult for me to live a life that I would value.”). The Chinese version of the scale showed acceptable reliability and structural validity [32].

#### 2.2.5. Sport Competition Anxiety Test, SCAT

The SCAT is a single-dimensional 15-item scale to measure the level of competitive trait anxiety in individuals (sample item: “I feel anxious before competition”). The scores are on 3-point Likert scale from 1 (“almost never”) to 3 (“very often”); the higher the total score, the higher the degree of trait anxiety in competitive situations. Zhu et al. (1993) translated and revised the SCAT scale into a Chinese version with acceptable reliability and validity [33].

Moreover, it is worth mentioning that all of questionnaire assessments in this study were presented in traditional Chinese.

#### 2.2.6. Shooting Performance Outcomes

(1) Free throw: Referring to the free-throw test method used in the study of Pojskić et al. (2014), athletes were tested for a group of 10 free throws with unlimited time, recording the number of free throws made. Each athlete was tested for only one group [34].
(2) Mid-range shot: Referring to the mid-range shot test method used in the study of Pojskić et al. (2014), athletes were tested for 20 mid-range shots 5 m away from basket—with the projection under the basket as the center, the radius is 5 m—at 5 shooting points (see Supplementary Materials). Four shots per shooting point were tested. Athletes can freely choose to start shooting from any shooting point. Each athlete was tested twice, and the researcher recorded the better number of shots made [34].
(3) Athletes were required to shoot outside the three-point line, and then rebound and dribble to shoot outside the three-point line again. One shooting session lasted 1 min. Each athlete was tested twice, and the researcher recorded the better number of shots made.

### 2.3. Data Analyses

The scales and data collected by this study were processed and analyzed using Microsoft Excel software and IBM SPSS 26(IBM Corp. in Armonk, New York, USA). The detailed methods for data analyses were as follows.

Descriptive statistics (means and standard deviations) were used to present the basic information about demographics (e.g., age, training years, etc.), psychological (e.g., trait anxiety, acceptance, etc.), and shooting performance outcomes. Independent-sample t-tests were used to analyze the between-group differences of psychological outcomes and shooting performance outcomes at baseline and 7-week post-test. The effect size was quantified by Cohen’s d. Paired-sample t-tests were to analyze the differences in psychological outcomes and shooting performance outcomes before and after the intervention. Multiple 2 × 2 repeated measures ANOVAs were used to investigate the differences in psychological skills and shooting performances. The effect size was quantified by Cohen’s d. The significance level was set *p* < 0.05 (two-tailed).

## 3. Results

The important demographics of the participants are presented in Table 2. As Table 2 shows, the intervention group and control group demonstrated no significant difference in age (t (41) = −0.79, *p* = 0.43), but they demonstrated significant difference in years of training (t (41) = 3.11, *p* = 0.003), as well as in training days per week (t (41) = 2.98, *p* = 0.005). These results further suggest that the intervention group previously accumulated longer training time than the control group did.

A series of independent-sample t-tests were conducted to compare psychological as well as shooting outcomes between the intervention group and control group at baseline. As the results in Table 3 showed, no significant difference in psychological and shooting outcomes was detected between the intervention group and control group.

To compare psychological as well as shooting outcomes between the intervention group and control group after intervention, independent-sample t-tests were also conducted. As the results in Table 4 showed, significant differences appeared in the total score of the FFMQ (t (41) = 2.83, *p* = 0.007, d = 0.88), the ‘describing’ dimension in the FFMQ (t (41) = 2.38, *p* = 0.022, d = 0.73), ‘nonreactivity to inner experience’ in the FFMQ (t (41) = 2.65, *p* = 0.012, d = 0.81), the total score of the AAQ-II (t (41) = −2.59, *p* = 0.013, d = 0.79), the score of the MAAS (t (41) = 2.20, *p* = 0.034, d = 0.67), the number of three-point shots (t (41) = 2.18, *p* = 0.035, d = 0.66), the number of three-pointers made (t (41)= 3.32, *p* = 0.002, d = 1.01), and the number of free throws made (t (41) = 2.82, *p* = 0.007, d = 0.86). All the other psychological and shooting outcomes did not reveal any significant differences.

A series of paired-sample t-tests were also conducted to compare psychological as well as shooting outcomes before and after intervention. As the results in Table 4 showed, significant differences appeared in the total score of the FFMQ (t (44) = 4.77, *p* = 0.000, d = 1.12), the ‘observing’ dimension in the FFMQ (t (44) = 2.38, *p* = 0.027, d = 0.57), the ‘describing’ dimension in the FFMQ (t (44) = 4.14, *p* = 0.000, d = 0.88), ‘nonreactivity to inner experience’ in the FFMQ (t (44) = 2.84, *p* = 0.010, d = 0.72), the total score of the AAQ-II (t (44)= −3.04, *p* = 0.006, d = 0.72), the score of the MAAS (t (44) = 2.90, *p* = 0.008, d = 0.63), the number of three-pointers made (t (44) = 3.01, *p* = 0.007, d = 0.57), and number of free throws made (t (44) = 3.24, *p* = 0.004, d = 0.69). All the other psychological and shooting outcomes did not reveal any significant differences.

As Table 5 showed, results from the repeated ANOVA demonstrated significant effect of interaction of time by intervention on the mindfulness (F = 11.128, *p* < *0*.01, η^2^ = 0.231) and mental flexibility reflected by AAQ-II (F = 7.417, *p*< 0.05, η^2^ = 0.167). The interaction of time with group had a significant effect on the total score of mindfulness (F = 13.616, *p* < *0*.01, η^2^ = 0.269), and attention (F = 5.169, *p* < *0*.01, η^2^ = 0.123). For the shooting performance, results showed that point-in-time interaction had significant effect on the number of three-pointers made (F = 8.935, *p* = 0.005, η^2^ = 0.195), the number of free throws made (F = 5.380, *p* = 0.026, η^2^ = 0.026). There was no significant interaction effect of age by time on psychological skills as well as shooting performances. There was also no significant three-way interaction (age, group, and time) between psychological skills and shooting performances.

## 4. Discussion

The use of mindfulness training in the field of sport psychology is growing and attracting the attention of a growing number of researchers. This unique study examined the effects of mindfulness-based intervention on college-level basketball athletes’ psychological outcomes and shooting performance. Overall, the intervention resulted in significant between-group and within-group differences in mindfulness level, acceptance level, attention level, three-point, and free-throw shooting performances (all *p*< 0.05). All of the significant between-group differences were found to have a medium to large effect size (Cohen’s d ranging from 0.565 to 1.117).

### 4.1. Intervention Effect on Psychological Outcomes

A key objective of the MAIC training developed for Chinese athletes is to enhance their psychological skills, particularly their mindfulness level. Before the intervention, there was no significant difference between the mindfulness level of the athletes between the intervention group and control group. After the intervention, it was found that the level of mindfulness, acceptance, and attention of the athletes in the intervention group was significantly higher than that of the control group athletes. Moreover, the results also showed that psychological outcomes in the intervention group significantly increased compared with baseline results, thus indicating that mindfulness training also resulted in significant within-group changes.

The enhancing effect of MAIC on mindfulness-related psychological outcomes is consistent with previous findings that mindfulness training can effectively improve athletes’ mindfulness level, emotional state, and psychological skills [15,16,35]. The researcher suggested that the reason MAIC reaps consistent success in improving psychological outcomes might be the addition of value and insight into the intervention program [15]. It has been argued that value and insight might be pivotal psychological constructs that lead to changes in a sequence of psychological outcomes such as action flexibility, value clarification, self-regulation, and exposure [36,37].

Regarding the enhancing effect of mindfulness intervention on other psychological outcomes such as attention and acceptance in the current study, the findings are similar to the updated evidence from Röthlin et al. (2020) [38], Kittler et al. (2022) [39], and Lu et al. (2021) [40], which found that mindfulness training improves athletes’ attention and increases awareness of the present moment. Moreover, it has been proven that the increased attention brought about by mindfulness training can lead to other positive effects such as a reduction in sport-related injury [41] and mental burnout [42].

Unexpectedly, the present study did not provide evidence that mindfulness intervention significantly relieved the sport performance anxiety level among collegiate basketball athletes. Previous research repeatedly demonstrated the positive emotion regulation effect (e.g., reduction of sport performance anxiety, fear, and stress, and improved flow status) [43,44,45,46]. The reason for the unexpected insignificant results might be the “floor effect” [47]. Specifically, since the collegiate basketball athletes recruited in the present study did not have much experience in participating in high-level competition, they reported very low levels of sport performance anxiety in the baseline test. Thus, the minimum baseline anxiety level of the participants creates a “floor” that results in measurement inaccuracy, as the further reduction of sport performance anxiety became nearly impossible.

### 4.2. Intervention Effect on Shooting Performance

The results in the present study revealed that mindfulness intervention can significantly improve collegiate basketball athletes’ three-point and free-throw performance. However, the mid-range shot performance was not significantly improved by intervention. This is because before making three-pointers and free throws, players have to prepare for the action before shooting, and most of the mid-range shots are in layups and fast breaks, so players do not have much time to prepare for the shot. Results from the previous study corroborated evidence that mindfulness training could significantly increase the free-throw percentage [20,21]. Moreover, the present study innovatively incorporated three-point shots into the shooting tests, to examine whether mindfulness training could improve three-point shot performance. As expected, the 7-week mindfulness training led to significant between-group and within-group differences in three-point shot performances. Steve Kerr revealed that the secret behind the high percentage of three-point shots among Golden State Warrior players is the additional mindfulness training [23]. The present study from an empirical angle corroborates that even a brief 7-week mindfulness training can significantly improve three-point shot performance. Due to the small sample size and convenience sampling, future study may enlarge the sample size and design the intervention more rigorously to further examine this positive effect. The present intervention did not significantly improve the mid-range shooting performance. Possible explanations could be that the athletes spent less time in training their mid-range shot compared to free-throw and three-point during their regular training time. The limited training time could be a possible explanation. Future research may thus investigate the interaction effect of mindfulness intervention with training time on mid-range shot performance.

### 4.3. Limitation

Through the 7-week experimental study, although significant positive changes appeared in some psychological outcomes and shooting performances, there were still some limitations to this study as follows. First, for practical reasons, the present study did not follow strict procedures (stratified sampling, perfect randomization, and double-blind) to conduct a more rigorous design such as a randomized controlled trial; thus, the internal and external validity of this study was impeded. Secondly, the sample size of the present study can be a potential limitation, and future study may consider enlarging the sample size to examine the effect of mindfulness training. Thirdly, for the statistical analyses, the present study employed multiple t-tests to examine the between-group and within-group differences, which might cause a possible type I error (false positive error); thus, the results from the current study might be explained with caution. Moreover, flaws in the current study also included but are not limited to: (1) insufficient information about socio-demographics such as no birth place or athletic level; (2) insufficient number of trials of the mid-range shot; (3) the lack of a pilot study for questionnaire validation leading to a floor effect in the reply; (4) the self-report questionnaire potentially negatively affecting the accuracy of the results due to social desirability and recall error.

## 5. Conclusions

While further study is necessary, the present study reveals that the 7-week mindfulness training program might enhance the level of mindfulness, acceptance, and attention as well as free-throw and three-point shooting accuracy in Macau collegiate basketball athletes. The present study provided supporting empirical evidence that even a brief 7-week mindfulness training program might result in positive psychological and performance changes. Future research, including real competitive high-pressure settings with neuroscience devices (such as electroencephalogram and functional magnetic resonance imaging), will broaden the scientific biological knowledge of how meditation affects the brain.

## Figures and Tables

**Table 1:** Content of MAIC intervention.

Week	Intervention Theme	Detailed Content
Week 1	Preparation of mindfulness training	Introduce the whole intervention program on mindfulness training, inform the athlete of the tasks in next 7 weeks, and arouse their enthusiasm for and interest in the upcoming intervention.
Week 2	Basic concepts in mindfulness training	Explain the basic concepts and skills in mindfulness training and encourage the athletes to experience this in light of their own examples.
Week 3	Self-decentralization training	Encourage athletes to shift from being self-oriented to task-oriented and present-focused.
Week 4	Acceptance training	Help athletes to improve their level of acceptance with no judgement and no over-reaction, accepting the being as it is.
Week 5	Value and insight training	Improve the athletes’ clarity about the direction of their behavior in the present moment.
Week 6	Commitment training	Anticipate and analyze the difficulties in the process of realizing values and learn how to overcome them and adhere to their own values.
Week 7	Comprehensive practice	Summarize the methods and skills learned in the intervention, conduct comprehensive exercises, and exchange experiences through communication.

**Table 2:** Comparison of demographic information of the participants by group.

	Intervention Group (*n* = 23)		Control Group (*n* = 20)		t	*p*
	Mean	SD	Mean	SD		
Age	20.74	1.71	21.20	2.09	−0.79	0.43
Years of training	8.00	2.92	5.35	2.62	3.11	0.003 **
Training days/week	4.39	1.08	3.55	0.69	2.98	0.005 **

Note: ** *p*< 0.01.

**Table 3:** Between-group difference at baseline.

Variable Names	Intervention Group (*n* = 23)	Control Group (*n* = 20)	*p*
Mindfulness			
FFMQ			
Observing	25.52 ± 5.31	24.90 ± 6.56	0.733
Describing	25.22 ± 4.00	27.80 ± 4.53	0.054
Acting with awareness	24.13 ± 5.51	27.85 ± 4.80	0.024
Nonjudging	22.17 ± 3.35	23.50 ± 4.27	0.261
Nonreactivity	21.52 ± 3.70	21.40 ± 4.25	0.920
Total score	118.57 ± 9.81	122.45 ± 11.17	0.231
MAAS			
Total score	3.78 ± 0.78	3.95 ± 0.91	0.512
Psychological Outcomes			
AAQ-II			
Total score	21.35 ± 7.33	23.55 ± 6.90	0.319
SCAT			
Total score	19.43 ± 3.29	19.85 ± 1.98	0.625
Shooting Performance			
Three-pointers made	5.30 ± 1.82	4.10 ± 2.13	0.052
Three-point attempts	11.09 ± 1.13	10.65 ± 0.81	0.157
Mid-range shots made	11.30 ± 2.91	10.05 ± 1.73	0.100
Free throws made	5.96 ± 1.97	5.75 ± 1.55	0.931

**Table 4:** Difference between baseline and post-test between two groups.

Variable Names	Group	Baseline	Post-Test	*p*	ES	∆	*p*
Mindfulness							
FFMQ							
Observing	Intervention group	25.52 ± 5.31	28.30 ± 4.48	0.027 *	0.566	2.78 ± 1.62	0.340
	Control group	24.90 ± 6.56	25.65 ± 6.21	0.684	0.117	0.75 ± 0.10	
Describing	Intervention group	25.22 ± 4.00	28.91 ± 4.40	0.000 ***	0.878	3.69 ± 4.28	0.001 ***
	Control group	27.80 ± 4.53	25.85 ± 3.96	0.137	0.458	−1.95 ± 5.62	
Acting with awareness	Intervention group	24.13 ± 5.51	26.48 ± 2.47	0.074	0.550	2.35 ± 2.00	0.039 *
	Control group	27.85 ± 4.80	26.25 ± 4.23	0.256	0.354	−1.60 ± 6.11	
Nonjudging	Intervention group	22.17 ± 3.35	23.39 ± 2.13	0.180	0.435	1.22 ± 4.22	0.229
	Control group	23.50 ± 4.27	22.90 ± 4.28	0.632	0.140	−0.60 ± 5.52	
Nonreactivity	Intervention group	21.52 ± 3.70	24.26 ± 3.95	0.010 *	0.716	2.74 ± 4.63	0.053
	Control group	21.40 ± 4.25	21.25 ± 3.45	0.891	0.039	−0.15 ± 4.85	
Total score	Intervention group	118.57 ± 9.81	131.35 ± 12.86	0.000 ***	1.117	12.78 ± 12.85	0.001 ***
	Control group	122.45 ± 11.17	121.90 ± 8.08	0.827	0.056	−0.55 ± 11.07	
MAAS							
Total score	Intervention group	3.78 ± 0.78	4.26 ± 0.75	0.008 **	0.627	0.48 ± 0.75	0.039 *
	Control group	3.95 ± 0.91	3.70 ± 0.92	0.623	0.541	−0.25 ± 1.42	
Psychological Outcomes							
AAQ-II							
Total score	Intervention group	21.35 ± 7.33	17.13 ± 3.76	0.006 **	0.724	−4.22 ± 6.66	0.616
	Control group	23.55 ± 6.90	20.45 ± 4.64	0.093	0.527	−3.10 ± 7.83	
SCAT							
Total score	Intervention group	19.43 ± 3.29	18.52 ± 4.50	0.165	0.231	−1.09 ± 3.63	0.306
	Control group	19.85 ± 1.98	19.90 ± 2.47	0.950	0.022	0.05 ± 3.53	
Shooting Performance							
Three-pointers made	Intervention group	5.30 ± 1.82	6.30 ± 1.72	0.007 **	0.565	1.00 ± 1.60	0.386
	Control group	4.10 ± 2.13	4.60 ± 1.64	0.309	0.263	0.50 ± 2.14	
Three-point attempts	Intervention group	11.09 ± 1.13	11.52 ± 0.90	0.076	0.421	0.43 ± 1.21	0.612
	Control group	10.65 ± 0.81	10.90 ± 0.97	0.383	0.280	0.25 ± 0.15	
Mid-range shots made	Intervention group	11.30 ± 2.91	11.00 ± 2.24	0.620	0.116	−0.30 ± 2.90	0.315
	Control group	10.05 ± 1.73	10.60 ± 1.98	0.350	0.296	0.55 ± 2.56	
Free throws made	Intervention group	5.96 ± 1.97	7.22 ± 1.68	0.004 **	0.688	1.26 ± 1.86	0.043 *
	Control group	5.75 ± 1.55	5.80 ± 1.61	0.909	0.032	0.05 ± 1.93	

Note: * *p* < 0.05; ** *p* < 0.01; *** *p* < 0.001; ES: effect size, Cohen’s d; ∆: mean difference calculated by post-test minus pre-test.

**Table 5:** Effect of mindfulness training on psychological skills and shooting performance with age differences.

Variable Names	Time			Time × Group			Time × Age			Time × Age × Group		
	F	*p*	η^2^	F	*p*	η^2^	F	*p*	η^2^	F	*p*	η^2^
Mindfulness												
FFMQ	11.128	0.002 **	0.231	13.616	0.001 **	0.269	0.631	0.538	0.033	1.668	0.203	0.083
MAAS	1.045	0.313	0.027	5.169	0.029 *	0.123	1.177	0.320	0.060	0.036	0.965	0.002
Psychological Outcomes												
AAQ-II	7.417	0.010 *	0.167	0.002	0.965	0.000	0.811	0.452	0.042	2.184	0.127	0.106
SCAT	0.257	0.615	0.007	0.185	0.670	0.005	0.288	0.752	0.015	1.231	0.304	0.062
Shooting Performance												
Three-pointers made	8.935	0.005 **	0.195	0.366	0.549	0.010	1.045	0.362	0.053	2.821	0.072	0.132
Three-point attempts	3.856	0.057	0.094	0.172	0.681	0.005	0.054	0.948	0.003	1.154	0.327	0.059
Mid-range shots made	0.658	0.422	0.017	0.458	0.503	0.012	0.210	0.811	0.011	2.037	0.145	0.099
Free throws made	5.380	0.026 *	0.127	3.481	0.070	0.086	0.411	0.666	0.022	0.574	0.568	0.030

Note: * *p*< 0.05; ** *p*< 0.01.

## Data Availability

Data are available by contacting the corresponding or first author.

## Supplementary Materials

The following supporting information can be downloaded at: https://www.mdpi.com/article/10.3390/ijerph20032339/s1, Figure S1: Mid-range Shot Positions.

## Abbreviations

PST: Psychological Skills Training; MAIC: Mindfulness–Acceptance–Insight–Commitment; FFMQ: Five-Facet Mindfulness Questionnaire; AAQ-II: Acceptance and Action Questionnaire—Second Edition; SCAT: Sport Competition Anxiety Test; MAAS: Mindful Attention Awareness Scale; ANOVA: Analysis of Variance.
