# Supplementary figures and images for: The Effect of Mindfulness Intervention on the Psychological Skills and Shooting Performances in Male Collegiate Basketball Athletes in Macau: A Quasi-Experimental Study
Source: Int J Environ Res Public Health. 2023 Jan 28;20(3):2339. doi: 10.3390/ijerph20032339 (PMC9916241; doi:10.3390/ijerph20032339)

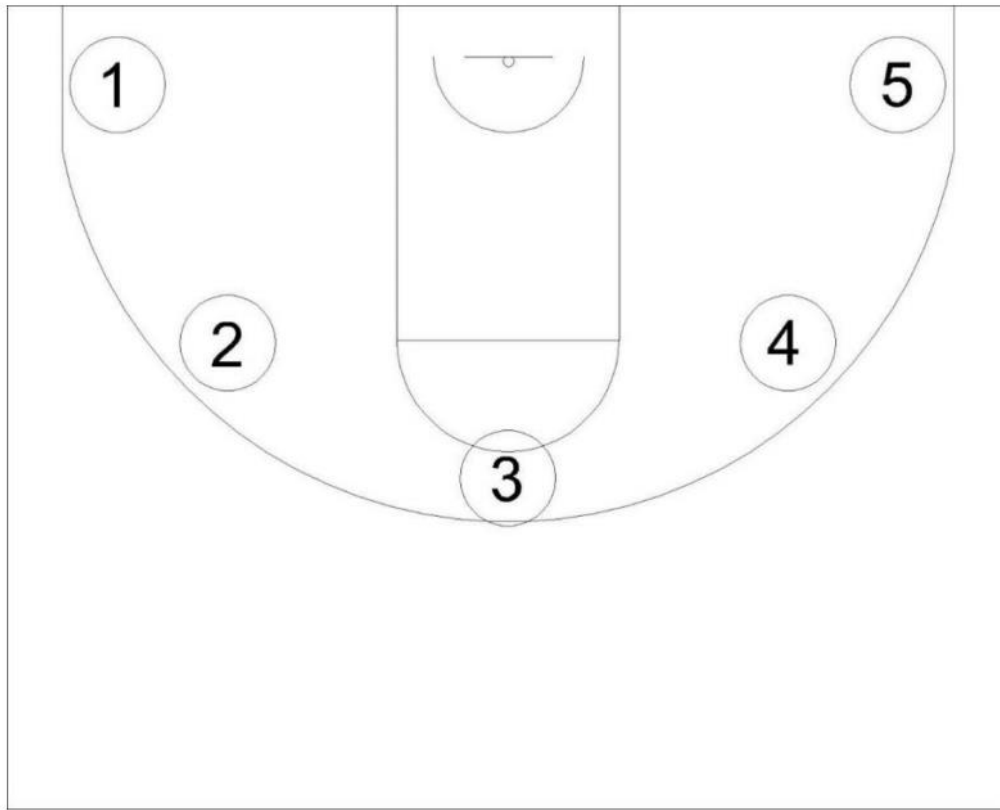

**Figure S1.** Mid-range Shot Positions.

Supplement: Supplementary file 1 [file ijerph-20-02339-s001.zip › ijerph-2093027-supplementary.pdf]
